# Comparison of seven comorbidity scores on four-month survival of lung cancer patients

**DOI:** 10.1186/s12874-023-01994-6

**Published:** 2023-11-03

**Authors:** Hélène Pluchart, Sébastien Bailly, Sébastien Chanoine, Denis Moro-Sibilot, Pierrick Bedouch, Anne-Claire Toffart

**Affiliations:** 1 Pôle Pharmacie, Centre Hospitalier Universitaire Grenoble Alpes, Grenoble, France; 2 Université Grenoble Alpes, Grenoble, France; 3 Univ. Grenoble Alpes, CNRS, Grenoble INP, Centre Hospitalier Universitaire Grenoble Alpes, TIMC UMR5525, Grenoble, 38000 France; 4 Univ. Grenoble Alpes, Inserm, Centre Hospitalier Universitaire Grenoble Alpes, HP2, Grenoble, 38000 France; 5 Institute for Advanced Biosciences, UGA/INSERM U1209/CNRS 5309, Université Grenoble Alpes, Grenoble, France; 6 Clinique de Pneumologie, Unité d’Oncologie Thoracique, Pôle Thorax et Vaisseaux, Centre Hospitalier Universitaire Grenoble Alpes, Grenoble, France

**Keywords:** Lung cancer, Comorbidity, Survival

## Abstract

**Background:**

The comorbidity burden has a negative impact on lung-cancer survival. Several comorbidity scores have been described and are currently used. The current challenge is to select the comorbidity score that best reflects their impact on survival. Here, we compared seven usable comorbidity scores (Charlson Comorbidity Index, Age adjusted Charlson Comorbidity Index, Charlson Comorbidity Index adapted to lung cancer, National Cancer Institute combined index, National Cancer Institute combined index adapted to lung cancer, Elixhauser score, and Elixhauser adapted to lung cancer) with coded administrative data according to the tenth revision of the International Statistical Classification of Diseases and Related Health Problems to select the best prognostic index for predicting four-month survival.

**Materials and methods:**

This cohort included every patient with a diagnosis of lung cancer hospitalized for the first time in the thoracic oncology unit of our institution between 2011 and 2015. The seven scores were calculated and used in a Cox regression method to model their association with four-month survival. Then, parameters to compare the relative goodness-of-fit among different models (Akaike Information Criteria, Bayesian Information Criteria), and discrimination parameters (the C-statistic and Harrell’s c-statistic) were calculated. A sensitivity analysis of these parameters was finally performed using a bootstrap method based on 1,000 samples.

**Results:**

In total, 633 patients were included. Male sex, histological type, metastatic status, CCI, CCI-lung, Elixhauser score, and Elixhauser-lung were associated with poorer four-month survival. The Elixhauser score had the lowest AIC and BIC and the highest c-statistic and Harrell’s c-statistic. These results were confirmed in the sensitivity analysis, in which these discrimination parameters for the Elixhauser score were significantly different from the other scores.

**Conclusions:**

Based on this cohort, the Elixhauser score is the best prognostic comorbidity score for predicting four-month survival for hospitalized lung cancer patients.

**Supplementary Information:**

The online version contains supplementary material available at 10.1186/s12874-023-01994-6.

## Background

The median age at lung cancer diagnosis is 70 years [1]. Given the increasing probability of developing comorbidities with age, the prevalence of comorbidity is higher in lung cancer than in other cancers, with at least 50 to 70% of patients having at least one comorbidity at diagnosis [2, 3].

The negative affect of comorbidities on patient survival are well described [4–6]. Since the development of the Charlson Comorbidity Index (CCI) [7], several comorbidity scores have been developed and are currently used. They all differ by the origin of the initial data source (administrative data or physician-reported data), their purpose (measuring comorbidity, measuring the impact of comorbidity and physical function), and comorbidity measures (organ or system-based approaches, counts of individual conditions and weighted indices) [8] (Table S1 Supplementary Materials 1). Some have been developed using lung cancer patient cohorts [9–11]. Despite the large number of comorbidity scores available, the CCI is the most studied and used comorbidity index in the medical literature [12, 13].

Certain comorbidity scores are based on the International Statistical Classification of Diseases and Related Health Problems (ICD-10), as Quan et al. published ICD-10 codes relative to comorbidities in 2005 [14]. They include the CCI (updated by Quan et al. in 2011 [7, 15]), CCI for lung cancer (named later CCI-lung) (Klabunde et al. in 2007) [16], age-adjusted CCI (ACCI) [17], Elixhauser score (updated in 2009 by Van Wallraven et al. [18, 19]), Elixhauser for lung cancer (Elixhauser-lung) (Mehta et al. [20]), National Cancer Institute Combined Index (NCI) [1], and NCI for lung cancer (NCI-lung) (Klabunde et al. in 2007 [16, 21]). They differ in terms of the type of comorbidities considered, the cohort used for validation, and their initial purpose [22].

Yang et al. found that the ACCI was better at predicting three-year overall survival than the CCI and Elixhauser score in a cohort of resected lung-cancer patients [23] based on administrative data coded using the ICD-9. However, they only compared the ACCI, CCI, and Elixhauser score. More recently, Mehta et al. proposed an Elixhauser score adapted to the cancer type (breast, lung, prostate, and colorectal). The cancer-specific Elixhauser score appears to be a better prognostic score for two-year survival than the cancer-specific NCI (developed by Klabunde et al.) [20].

Although the CCI is the most widely used comorbidity score, it would be informative to assess which score is more predictive of mortality in cohorts with administrative data. Here, we compared the seven comorbidity scores available using administrative data coded using the ICD-10 in predicting four-month survival of our cohort of hospitalized lung-cancer patients.

## Materials and methods

### Data source and population

We included patients hospitalized in the Thoracic Oncology Unit of Grenoble Alpes University Hospital from 2011 to 2015 described in an earlier publication [24]. Lung-cancer patients were included at their first hospitalization during the studied period.

The study was approved by our institutional review board and ethics approval was obtained on September 1, 2021 (CECIC Rhône-Alpes-Auvergne, Clermont-Ferrand, IRB 5891).

The database contains information on individuals including their age, gender, lung cancer’ TNM staging, performance status at their first presentation case in multidisciplinary concertation meetings, and the histological type of the lung cancer.

### Outcome and covariates

The outcome was median overall survival. Survival data were obtained from our district cancer registry, including the date of the last follow-up and the vital status at the last follow-up. Right censored date point was defined by median overall survival.

Age, gender, lung cancer metastatic status, histologic type, age at hospitalization, and age at diagnosis were included as covariates.

### Comorbidity scores

Data concerning comorbidities were obtained by the Health Information Services Department and coded using the ICD-10. The diagnoses for comorbidities were recorded at the patients’ discharge in our medical unit. Seven comorbidity scores were calculated: CCI, ACCI, CCI-lung, NCI, NCI-lung, Elixhauser, and Elixhauser-lung. We did not record metastatic solid tumors and lung cancer as comorbid conditions. The seven scores are summarized in Table 1.

**Table 1:** Summary of differences between the seven comorbidity scores used

Score	Number of conditions	Scoring method	Score range
**Updated CCI** (15)	12 conditions	Based on 1-year-all-cause mortality Sum of weighted indices (derived based on hazards ratios) Updated by Quan et al. (15)	0 to 24
**CCI-lung** (16)	10 conditions	Based on the impact on 2-year non-cancer mortality Sum of weighted indices (derived based on hazards ratios)	0 to 15
**ACCI** (17)	12 conditions	Based on 1-year-all-cause mortality Age-adjusted CCI is equal to the CCI score but 1 point has to be added for each decade above 50 years	CCI + 1 point added for each decade above 50 years old
**NCI** (1)	14 conditions	Based on the impact on 2-yr non-cancer mortality Sum of weighted indices (derived from hazards ratios, available on the NCI website)	0 to 21.14
**NCI-lung** (16)	13 conditions	Based on the impact on 2-yr non-cancer mortality Sum of weighted indices (derived from beta coefficients)	-0.143 to 4.243
**Updated Elixhauser** (19)	21 conditions	Used initially as a count (30 conditions) but modified by Van Walraven et al. Based on in-hospital mortality as the sum of the weighted score (hazards ratios derived from beta coefficients divided by the coefficient in the model with the smallest absolute value) (19)	-19 to 89
**Elixhauser-lung** (20)	16 conditions	Based on the impact on 2-yr non-cancer mortality Sum of the weighted indices (derived from the beta coefficient x 10)	-2 to 28

### Statistical analyses

For descriptive analysis, quantitative variables are expressed as medians [Interquartile ranges] and qualitative variables as n (%).

Comorbidity scores were calculated and survival estimated as the time between the day of hospitalization and the date of last follow-up (cut off at cohort’s estimated median overall survival which was our right censored date point). The Kaplan Meier estimator was used to estimate the probability of survival. Log-rank tests were used to compare the probability of the event (death) between populations. The model was adjusted for each score. A Cox proportional hazards regression model was used to perform multivariable analyses of prognostic factors and calculate hazard ratios (HRs) and 95% confidence intervals (95% CI) for median survival for the seven comorbidity scores. A median cut-off was used for continuous variables in the multivariate analysis. Proportional hazards assumptions were verified using the Martingale method [25]. Only covariables with a p-value < 0.2 were retained for multivariable analysis.

To compare comorbidity scores, Akaike Information Criteria (AIC), Bayesian Information Criteria (BIC) were used to compare the relative goodness-of-fit among different models. Then, a discrimination analysis using the c-statistic, Harrell’s c-statistic, sensitivity, specificity was performed from a base model containing significant covariables from the multivariate analysis. Sensitivity and specificity were respectively calculated as follow: (True positive = Death estimated by the model) / (True positive + false negative (= patient estimated as non-dead by the model although they are dead)); and (true negative = non-dead patients estimated by the model) / (true negative + false positive = estimated dead by the model although they are not).

The impact of the scores was compared using the base model (significant covariables in multivariable analysis) plus each index score alone by multivariable Cox regression. The model with the lowest AIC and BIC indicates which model was the best fit for the data and the highest c statistic and Harrell’s c statistic was considered to be the best predictive model.

A sensitivity analysis using a bootstrap method for each statistical indicator, with 1,000 samples from two thirds of the cohort, was performed. Each indicator (AIC, BIC, c-statistic, and Harrell’s c-statistic) was calculated for the 1,000 samples. Boxplots were generated for the four parameters.

All statistical analyses were performed using SAS 9.4 for Windows (SAS Institute, Inc., Cary, NC, USA). A p-value < 0.05 was considered significant.

## Results

### Descriptive analyses and adjusted hazard ratios for four-month survival using the seven comorbidity scores

In total, 633 patients were enrolled in the study. The demographic characteristics of the population are presented in Table 2. The median age [IQ25%;IQ75%] at diagnosis was 65 [58–72] years and 540 (71%) of the patients were men. The median survival from hospital admission was 4 [1; 11] months. A Kaplan Meier curve of follow-up time (which corresponds to survival) in this cohort (cut off at 4 months as 4 months was median overall survival) is represented in supplementary materials (Figure S1). Among the cohort, 428 patients (74%) had metastatic lung cancer and 295 (47%) had adenocarcinoma. The diagnosis of cancer was made before hospitalization for most of the patients (518, 82%).

**Table 2:** Baseline characteristics and adjusted hazard ratios of four-month survival in the cohort (n = 633)

Population (n = 633)		Univariate analysis		Multivariate analysis		
Characteristics		HR [95% CI]	P value	HR [95% CI]	P value	Type 3 P Value
Overall survival, months	4 [1; 11]					
Length of stay in the hospital (days)	11 [6; 20]					
Performance status at the first case presentation in multidisciplinary concertation meetings*						
PS 0–1	212 (53.1)					
PS 2	126 (31.6)					
PS 3–4	61 (15.3)					
Men	540 (71.0)	1.3 [1.0; 1.6]	0.07	1.3 [1.0; 1.7]	0.03	
Metastasis	428 (74.2)	2.0 [1.5; 2.6]	< 0.01	1.9 [1.3; 2.9]	< 0.01	
Histological type						< 0.01
Adenocarcinoma	295 (46.6)	1		1		
Squamous-cell carcinoma	103 (16.3)	0.9 [0.6; 1.2]	0.40	1.0 [0.7; 1.4]	0.87	
Undifferentiated carcinoma	67 (10.6)	0.4 [1.2; 0.8]	0.40	1.2 [0.8; 1.7]	0.33	
Small-cell lung cancer	132 (20.9)	0.7 [0.5; 0.9]	< 0.01	0.7 [0.5; 0.9]	< 0.01	
Other types	36 (5.7)	1.9 [1.2; 2.8]	< 0.01	1.9 [1.3; 2.9]	< 0.01	
Age during hospitalization	66 [58;73]	1.1 [0.9; 1.3]	0.54			
Age at diagnostic	65 [58;72]	1.1 [0.9; 1.3]	0.60			
Time of cancer diagnosis						
Diagnosis before hospitalization	518 (81.8)	1				
Diagnosis during or after hospitalization	115 (18.2)	0.9 [0.7; 1.2]	0.47			

*Quantitative variables are expressed as medians [Interquartile range], qualitative variables are expressed as n (%)*

*Missing data: n = 234

In multivariable analysis, only the presence of metastases, male gender, and histological type were prognostic factors of four-month survival.

We assessed the prevalence of each comorbidity that contributed to the score for each comorbidity score (Table 3 for the Elixhauser score and Elixhauser-lung and Tables S2 and S3 in Supplementary Materials for CCI, CCI-lung, NCI, and NCI-lung). For the Elixhauser score, weight loss, fluid and electrolyte disorders, and chronic pulmonary disease were the three most common comorbidities, whereas weight loss, chronic pulmonary disease, and peripheral vascular disease were the most common comorbidities for Elixhauser-lung. The common thread between the Elixhauser score, Elixhauser-lung, CCI, CCI-lung, NCI, and NCI-lung was the high prevalence of chronic pulmonary disease, which was among the three most common comorbidities in the cohort.

**Table 3:** Description of comorbidity in the population according to the Elixhauser score and Elixhauser-lung

Population (n = 633)	Elixhauser	Elixhauser-lung
Congestive Heart Failure	46 (7.3)	46 (7.3)
Cardiac arrythmias	77 (12.2)	
Vascular disease	22 (3.5)	22 (3.5)
Pulmonary circulation disorders	40 (6.3)	40 (6.3)
Peripheral vascular disorders	48 (7.6)	48 (7.6)
Paralysis	46 (7.3)	46 (7.3)
Neurodegenerative disorders	42 (6.6)	42 (6.6)
Chronic pulmonary disease	91 (14.4)	91 (14.4)
Renal failure	26 (4.1)	26 (4.1)
Liver disease	19 (3.0)	
Lymphoma	4 (0.6)	
Cancer*	65 (10.3)	
Coagulopathy	27 (4.3)	27 (4.3)
Obesity	11 (1.7)	11 (1.7)
Weight loss	345 (54.5)	345 (54.5)
Fluid and electrolyte disorders	91 (14.4)	
Blood loss anemia	6 (1.0)	
Deficiency anemia	14 (2.2)	
Drug abuse	8 (1.3)	
Depression	29 (4.6)	29 (4.6)
Diabetes with complications		11 (1.7)
Hypothyroidism		17 (2.7)
Rheumatological disorders		4 (0.6)
Psychosis		9 (1.4)

*Qualitative variables are expressed as n (%)*

*excluding lung cancer and metastatic solid tumor

Among the seven scores, in terms of the p value and type 3 p value, an ACCI ≥ 5, Elixhauser score > 11, and Elixhauser-lung ≥ 4 were associated with lower survival. However, neither the CCI, CCI-lung, NCI, nor NCI-lung were associated with poorer survival (Table 4).

**Table 4:** Adjusted hazard ratios for four-month survival among the population (n = 633)

Comorbidity score	Median [Interquartile range]	HR [95% CI]	P value	Type 3 P value
CCI	0.0 [0.0; 2.0]			0.1
0		1		
1 or 2		1.1 [0.8; 1.4]	0.63	
≥ 3		1.4 [1.0; 2.1]	0.04	
ACCI*	3.0 [2.0; 4.0]			**< 0.05**
≤ 2		1		
3		1.0 [0.8; 1.4]	0.88	
4		1.3 [0.9; 1.7]	0.13	
≥ 5		**1.6 [1.1; 2.1]**	**< 0.01**	
CCI-lung	0.0 [0.0; 1.0]			0.08
0		1		
1 ≤ CCI-lung ≤ 2		1.1 [0.9; 1.5]	0.31	
≥ 3		**1.5 [1.0; 2.2]**	**0.03**	
NCI*	0.0 [0.0; 1.7]			0.47
0 < NCI ≤ 1		1		
1 < NCI ≤ 2		1.2 [0.9; 1.5]	0.30	
2 < NCI < 3		1.1 [0.7; 1.7]	0.63	
NCI ≥ 3		1.3 [0.9; 1.8]	0.16	
NCI-lung	0.0 [0.0; 0.33]			0.22
< 0		1		
0 = NCI ≤ 0.2		1.3 [0.7; 2.3]	0.50	
0.2 < NCI ≤ 0.4		1.6 [0.8; 3.2]	0.14	
NCI > 0.4		1.4 [0.8; 2.7]	0.26	
Elixhauser	6.0 [2.0; 11.0]			**< 0.01**
Elixhauser ≤ 0 to Elixhauser ≤ 5		1		
6 ≤ Elixhauser ≤ 11		1.4 [1.0; 1.8]	0.02	
Elixhauser > 11		**1.6 [1.2; 2.1]**	**< 0.01**	
Elixhauser-lung*	3.0 [0.0; 5.0]			**< 0.01**
≤ 0		1		
1 ≤ Elixhauser-lung ≤ 3		1.2 [0.9; 1.7]	0.14	
4 ≤ Elixhauser-lung ≤ 5		**2.1 [1.5; 3.0]**	**< 0.01**	
Elixhauser-lung > 5		**1.5 [1.1; 2.0]**	**0.02**	

*Adjusted for sex, presence of metastases, and histological type. Quantitative variables are expressed as medians [Interquartile range]; In bold: p < 0.05*

*sex is no longer significantly different in multivariate analysis with this score

### Model comparison and discrimination analyses between the seven scores and the bootstrap method

We calculated the AIC, BIC, c-statistic, Harrell’s c-statistic, sensitivity and specificity (Table 5). The Elixhauser score had the lowest AIC and BIC. It also had highest c-statistic and Harrell’s c-statistic as discriminative parameters, indicating that this score is the best predictive model for estimating four-month survival in our cohort.

**Table 5:** Model comparison with AIC, BIC and discrimination parameters between the seven comorbidity scores

	AIC	BIC	Harrell’s c-statistic	C-statistic	Sensitivity (%)	Specificity (%)
Base model*	4755.8721	4758.0284	0.6141	0.649	62.84	59.68
Base model + CCI	4753.5483	4755.7528	0.6189	0.657	61.34	61.88
Base model + ACCI	4745.7800	4747.9845	0.6249	0.663	63.23	61.99
Base model + CCI-lung	4752.0853	4754.2898	0.6209	0.658	61.27	61.95
Base model + NCI	4751.9881	4754.1925	0.6194	0.656	62.69	59.52
Base model + NCI-lung	4749.9826	4752.1871	0.6236	0.658	60.90	61.37
Base model + Elixhauser	**4744.7953**	**4746.9998**	**0.6314**	**0.673**	**64.66**	**62.57**
Base model+ Elixhauser-lung	4749.5804	4751.7849	0.6251	0.669	63.84	61.05

*Adjusted for sex, metastasis, and histological type

We confirmed this trend by generating boxplots from the sensitivity analyses of the AIC, BIC, c-statistics, and Harrell’s c-statistic (Supplementary Figures S2).

## Discussion

The CCI has been shown to be associated with poorer survival for all TNM stage lung-cancer patients [12, 26] and is the most widely used comorbidity score. Here, we performed the first study to compare seven comorbidity scores on a cohort of lung-cancer patients. Sarfati et al. suggested that the CCI, cancer-specific NCI, and Elixhauser score may be the preferred comorbidity scores when using administrative data [22]. In this study, we used the ICD-10 to identify comorbidity from administrative data and found the Elixhauser score to be the best score for predicting four-month mortality.

The Elixhauser score has already been compared to the CCI for patients with cancers other than lung cancer and found to be a better prognosis score for colorectal and oral cancer patients [27, 28]. Mehta et al. found that the lung cancer-specific Elixhauser performed better than the lung cancer-specific NCI and Elixhauser score [29]. The outcome of the aforementioned study was two-year non-cancer mortality, which had a consequence on the statistical analyses because the authors had to consider competing risks. In addition, they studied comorbidities prior to the lung cancer diagnosis. More interestingly, they also compared these scores to the individual Charlson and Elixhauser comorbidity scores. The Elixhauser individual comorbidity scores performed better than the Charlson individual comorbidity scores. However, scores have been shown to be good substitutes for individual comorbidity variables in health services research [30]. In a paper published by Yang et al., the ACCI predicted overall three-year survival better than the CCI or Elixhauser score [23]. In contrast to Mehta et al., they did not discriminate between death from cancer and other causes, but they did consider comorbidities prior to the diagnosis of lung cancer.

These scores differ not only in the way they were constructed (origin of the cohort and outcome chosen), but also in the weight assigned to each comorbidity; some use the beta coefficient obtained from the regression and others the hazard ratio. The beta coefficients and hazard ratios are related to each other by an exponential relationship, and although the use of beta coefficients is preferred when using a summary score [31], we calculated the comorbidity scores as they were described and published in the original papers.

There are several possible explanations concerning the better performance of the Elixhauser score. The Elixhauser score was developed using a short-term outcome: in-hospital mortality. The median overall survival in our cohort was four months, which is short relative to the other scores (i.e., the CCI), which were constructed using a long-term outcome, such as one- or two-year mortality. This result corroborates another publication concerning in-hospital mortality of non-cancer patients, in which the Elixhauser score outperformed the CCI [32]. Another possibility is the number of comorbidities taken into account in the Elixhauser score, which is more than for the other scores. Lung cancer patients have the most comorbidities at diagnosis relative to patients with other types of cancer, especially due to tobacco exposure [3, 33]. This could explain why the Elixhauser score best fit our cohort in predicting four-month mortality.

This study had several limitations. We assessed comorbidities that occurred both before and after lung cancer diagnosis and did not distinguish between death from lung cancer and that from other causes. Extension of this paper results should be done with one caution. Despite we had 71% of men and 74.2% of patients with metastatic status at diagnosis, which is similar to literature, age have a non-significant effect on survival. This may be due to the inclusion criteria which is hospitalized patients and therefore frailty ones with the shortest survivals, and high comorbidity burden (Median Elixhauser score of 6). Because performance test has been performed on the same data used to train the model there will be a need for external validity of the results. There may have also been unknown confounders. Moreover, this was a retrospective monocentric study.

The use of ICD-10 codes to identify the comorbidities was a strength of our study, as they can be used to query easily available structured datasets and allow the comparison of comorbidity scores, as well as sensitivity analyses, which confirmed the superiority of the Elixhauser score for estimating four-month survival in our cohort.

## Conclusions

Despite the extensive use of the CCI in the literature, other comorbidity scores are available, including scores based on administrative data coded using the ICD-10. In this original study, in which we compared seven comorbidity scores using administrative data, the Elixhauser score was the comorbidity score best suited to hospitalized lung-cancer patients for predicting four-month mortality. It could be informative to repeat these analyses with a longer follow-up of the patients.

### Electronic supplementary material

Below is the link to the electronic supplementary material.

Supplementary Material 1: Table S1

Supplementary Material 2: Figure S1

Supplementary Material 3: Table S2

Supplementary Material 4: Table S3

Supplementary Material 5: Figure S2

## Data Availability

The datasets generated and/or analysed during the current study are not publicly available due to privacy restrictions but are available from the corresponding author on reasonable request. The description of their content and how it has been obtained is described in the protocol cited above [24].

## Abbreviations

ACCI: Age-adapted Charlson Comorbidity Index
AIC: Akaike Information Criteria
BIC: Bayesian Information Criteria
CCI: Charlson Comorbidity Index
CCI-lung: Charlson Comorbidity Index adapted to lung cancer
CIRS: Cumulative Illness Rating Scale
CIRS-G: Cumulative Illness Rating Scale adapted for geriatric population
Elixhauser: Elixhauser score
Elixhauser-lung: Elixhauser score adapted to lung cancer
KFI: Kaplain Feinstein Index
NCI: National Cancer Institute combined index
NCI-lung: National Cancer Institute combined index adapted to lung cancer
SCI: Simplified Comorbidity Index

## References

[1] Noone AM, Howlader N, Krapcho M, Miller D, Brest A, Yu M et al. SEER Cancer Statistics Review, 1975–2015, National Cancer Institute [Internet]. [cited 2018 Jul 30]. Available from: https://seer.cancer.gov/csr/1975_2015/sections.html.

[2] Edwards BK, Noone AM, Mariotto AB, Simard EP, Boscoe FP, Henley SJ et al. Annual Report to the Nation on the status of cancer, 1975–2010, featuring prevalence of comorbidity and impact on survival among persons with lung, colorectal, breast, or prostate cancer. Cancer. 2014;120(9):1290–314. 10.1002/cncr.28509 PMC3999205 24343171

[3] Islam KMM, Jiang X, Anggondowati T, Lin G, Ganti AK (2015). Comorbidity and Survival in Lung Cancer Patients. Cancer Epidemiol Biomark Prev Publ Am Assoc Cancer Res Cosponsored Am Soc Prev Oncol.

[4] Moro-Sibilot D, Aubert A, Diab S, Lantuejoul S, Fourneret P, Brambilla E (2005). Comorbidities and Charlson score in resected stage I nonsmall cell lung cancer. Eur Respir J.

[5] Seigneurin A, Delafosse P, Trétarre B, Woronoff AS, Velten M, Grosclaude P et al. Are comorbidities associated with long-term survival of lung cancer? A population-based cohort study from French cancer registries. BMC Cancer [Internet]. 2018 Dec [cited 2019 Feb 13];18(1). Available from: https://bmccancer.biomedcentral.com/articles/10.1186/s12885-018-5000-7. 10.1186/s12885-018-5000-7 PMC6233579 30419850

[6] Leduc C, Antoni D, Charloux A, Falcoz PE, Quoix E. Comorbidities in the management of patients with lung cancer. Eur Respir J. 2017;49(3). 10.1183/13993003.01721-2016 28356370

[7] Charlson ME, Pompei P, Ales KL, MacKenzie CR (1987). A new method of classifying prognostic comorbidity in longitudinal studies: development and validation. J Chronic Dis.

[8] Sarfati D (2012). Review of methods used to measure comorbidity in cancer populations: no gold standard exists. J Clin Epidemiol.

[9] Tammemagi CM (2005). Comorbidity and Survival Disparities among Black and White patients with breast Cancer. JAMA.

[10] Tammemagi CM, Neslund-Dudas C, Simoff M, Kvale P (2003). Impact of comorbidity on lung cancer survival. Int J Cancer.

[11] Colinet B, Jacot W, Bertrand D, Lacombe S, Bozonnat MC, Daurès JP (2005). A new simplified comorbidity score as a prognostic factor in non-small-cell lung cancer patients: description and comparison with the Charlson’s index. Br J Cancer.

[12] Moro-Sibilot D, Aubert A, Diab S, Lantuejoul S, Fourneret P, Brambilla E (2005). Comorbidities and Charlson score in resected stage I nonsmall cell lung cancer. Eur Respir J.

[13] Bannay A, Chaignot C, Blotière PO, Basson M, Weill A, Ricordeau P (2016). The best use of the Charlson Comorbidity Index with Electronic Health Care Database to Predict Mortality. Med Care.

[14] Quan H, Sundararajan V, Halfon P, Fong A, Burnand B, Luthi JC (2005). Coding algorithms for defining comorbidities in ICD-9-CM and ICD-10 administrative data. Med Care.

[15] Quan H, Li B, Couris CM, Fushimi K, Graham P, Hider P (2011). Updating and validating the Charlson Comorbidity Index and score for Risk Adjustment in Hospital discharge abstracts using data from 6 countries. Am J Epidemiol.

[16] Klabunde CN, Legler JM, Warren JL, Baldwin LM, Schrag D (2007). A refined comorbidity measurement algorithm for claims-based studies of breast, prostate, colorectal, and lung cancer patients. Ann Epidemiol.

[17] Charlson M, Szatrowski TP, Peterson J, Gold J (1994). Validation of a combined comorbidity index. J Clin Epidemiol.

[18] Elixhauser A, Steiner C, Harris DR, Coffey RM (1998). Comorbidity measures for use with administrative data. Med Care.

[19] van Walraven C, Austin PC, Jennings A, Quan H, Forster AJ (2009). A modification of the Elixhauser comorbidity measures into a point system for hospital death using administrative data. Med Care.

[20] Mehta HB, Sura SD, Adhikari D, Andersen CR, Williams SB, Senagore AJ (2018). Adapting the Elixhauser comorbidity index for cancer patients. Cancer.

[21] Klabunde CN, Potosky AL, Legler JM, Warren JL (2000). Development of a comorbidity index using physician claims data. J Clin Epidemiol.

[22] Sarfati D (2012). Review of methods used to measure comorbidity in cancer populations: no gold standard exists. J Clin Epidemiol.

[23] Yang CC, Fong Y, Lin LC, Que J, Ting WC, Chang CL (2018). The age-adjusted Charlson comorbidity index is a better predictor of survival in operated lung cancer patients than the Charlson and Elixhauser comorbidity indices. Eur J Cardiothorac Surg.

[24] Pluchart H, Bailly S, Fauconnier J, Delafosse P, Chanoine S, Dumas I et al. Study protocol to assess polypharmacy and comorbidities in lung cancer. Respir Med Res. 2021;100861. 10.1016/j.resmer.2021.100861 34662777

[25] Therneau TM, Grambsch PM, Fleming TR (1990). Martingale-based residuals for survival models. Biometrika.

[26] Zhao L, Leung LH, Wang J, Li H, Che J, Liu L (2017). Association between Charlson comorbidity index score and outcome in patients with stage IIIB-IV non-small cell lung cancer. BMC Pulm Med.

[27] Lieffers JR, Baracos VE, Winget M, Fassbender K (2011). A comparison of charlson and elixhauser comorbidity measures to predict colorectal cancer survival using administrative health data. Cancer.

[28] Chang HJ, Chen PC, Yang CC, Su YC, Lee CC. Comparison of Elixhauser and Charlson Methods for Predicting oral Cancer survival: Medicine (Baltimore). 2016;95(7):e2861. 10.1097/MD.0000000000002861 PMC4998653 26886653

[29] Mehta HB, Sura SD, Adhikari D, Andersen CR, Williams SB, Senagore AJ (2018). Adapting the Elixhauser comorbidity index for cancer patients. Cancer.

[30] Austin SR, Wong YN, Uzzo RG, Beck JR, Egleston BL (2015). Why Summary Comorbidity Measures such as the Charlson Comorbidity Index and Elixhauser score work. Med Care.

[31] Mehta HB, Mehta V, Girman CJ, Adhikari D, Johnson ML (2016). Regression coefficient–based scoring system should be used to assign weights to the risk index. J Clin Epidemiol.

[32] Sharma N, Schwendimann R, Endrich O, Ausserhofer D, Simon M (2021). Comparing Charlson and Elixhauser comorbidity indices with different weightings to predict in-hospital mortality: an analysis of national inpatient data. BMC Health Serv Res.

[33] Edwards BK, Noone AM, Mariotto AB, Simard EP, Boscoe FP, Henley SJ et al. Annual Report to the Nation on the status of cancer, 1975–2010, featuring prevalence of comorbidity and impact on survival among persons with lung, colorectal, breast, or prostate cancer. Cancer. 2014;120(9):1290–314. 10.1002/cncr.28509 PMC3999205 24343171

