# Supplementary material for: Comparison of seven comorbidity scores on four-month survival of lung cancer patients
Source: BMC Med Res Methodol. 2023 Nov 3;23:256. doi: 10.1186/s12874-023-01994-6 (PMC10623755; doi:10.1186/s12874-023-01994-6)
Supplement: Supplementary file 1 — Supplementary Material 1: Table S1 [file 12874_2023_1994_MOESM1_ESM.docx]

| Score | Year of publication | Initial data source | System or condition on which it is based and the number of items | Population | Scoring method | Examples of references in lung cancer after develop-ment of the score |
| --- | --- | --- | --- | --- | --- | --- |
| Kaplain Feinstein Index (KFI) [1] | 1974 | Clinical notes | 12 systems | 188 patients with diabetes | Highest score of single item | [2,3] |
| Cumulative Illness Rating Scale (CIRS) [4] and CIRS adapted for geriatric population (CIRS-G) [5] | 1968 | Clinical notes | 13 or 14 systems | Not available | Sum of systems concerned | [6–9] |
| Charlson Comorbidity Index score (CCI) [10] | 1987 | Clinical notes | 17 conditions | 604 patients in general medical units | Sum of weighted indices (weights being equivalent to the (rounded) adjusted relative risks for 1-year mortality for each condition  Updated by Quan et al. [11]  Age-adjusted CCI is equal to the CCI score but 1 point has to be added for each decade above 50 years [12] | [13–20] |
| Elixhauser [21] | 1998 | Administrative data | 30 conditions | 1,779,167 adults in acute  care hospital | Used initially as a count but modified by Van Walraven et al. as a weighted score [22] for in-hospital death | [23] |
| Tammemagi [24,25] | 2003 | Administrative data | 19 conditions for lung cancer and 77 conditions for breast cancer | 1155 patients with lung cancer and 906 patients with breast cancer | Condition count | / |
| Simplified Comorbidity  Index (SCI) [26] | 2005 | Clinical notes | 7 comorbidity categories | 735 patients with lung cancer | Impact on mortality (beta coefficients from multivariable models). | [27–30] |

**Table S1.** Characteristics of comorbidity scores to assess their burden on survival used in the literature in lung cancer, adapted from [31]

**References for table S1**

1. Kaplan MH, Feinstein AR. The importance of classifying initial co-morbidity in evaluating the outcome of diabetes mellitus. J Chronic Dis. 1974 Sep;27(7–8):387–404.

2. Maestu I, Muñoz J, Gómez-Aldaraví L, Esquerdo G, Yubero A, Torregrosa MD, et al. Assessment of functional status, symptoms and comorbidity in elderly patients with advanced non-small-cell lung cancer (NSCLC) treated with gemcitabine and vinorelbine. Clin Transl Oncol. 2007 Feb;9(2):99–105.

3. Battafarano RJ, Piccirillo JF, Meyers BF, Hsu HS, Guthrie TJ, Cooper JD, et al. Impact of comorbidity on survival after surgical resection in patients with stage I non–small cell lung cancer. J Thorac Cardiovasc Surg. 2002 Feb;123(2):280–7.

4. Linn BS, Linn MW, Gurel L. Cumulative illness rating scale. J Am Geriatr Soc. 1968 May;16(5):622–6.

5. Parmelee PA, Thuras PD, Katz IR, Lawton MP. Validation of the Cumulative Illness Rating Scale in a Geriatric Residential Population. J Am Geriatr Soc. 1995 Feb;43(2):130–7.

6. Kirkhus L, Jordhøy M, Šaltytė Benth J, Rostoft S, Selbæk G, Jensen Hjermstad M, et al. Comparing comorbidity scales: Attending physician score versus the Cumulative Illness Rating Scale for Geriatrics. J Geriatr Oncol. 2016 Mar;7(2):90–8.

7. Grønberg BH, Valan CD, Halvorsen T, Sjøblom B, Jordhøy MS. Associations between severe co‐morbidity and muscle measures in advanced non‐small cell lung cancer patients. J Cachexia Sarcopenia Muscle. 2019 Dec;10(6):1347–55.

8. Baker S, Sharma A, Peric R, Heemsbergen WD, Nuyttens JJ. Prediction of early mortality following stereotactic body radiotherapy for peripheral early-stage lung cancer. Acta Oncol. 2019 Feb 1;58(2):237–42.

9. Firat S, Byhardt RW, Gore E. The Effects of Comorbidity and Age on RTOG Study Enrollment in Stage III Non–Small Cell Lung Cancer Patients Who Are Eligible for RTOG Studies. Int J Radiat Oncol. 2010 Dec;78(5):1394–9.

10. Charlson ME, Pompei P, Ales KL, MacKenzie CR. A new method of classifying prognostic comorbidity in longitudinal studies: Development and validation. J Chronic Dis. 1987 Jan;40(5):373–83.

11. Quan H, Li B, Couris CM, Fushimi K, Graham P, Hider P, et al. Updating and Validating the Charlson Comorbidity Index and Score for Risk Adjustment in Hospital Discharge Abstracts Using Data From 6 Countries. Am J Epidemiol. 2011 Mar 15;173(6):676–82.

12. Charlson M, Szatrowski TP, Peterson J, Gold J. Validation of a combined comorbidity index. J Clin Epidemiol. 1994 Nov;47(11):1245–51.

13. Islam KMM, Jiang X, Anggondowati T, Lin G, Ganti AK. Comorbidity and Survival in Lung Cancer Patients. Cancer Epidemiol Biomark Prev Publ Am Assoc Cancer Res Cosponsored Am Soc Prev Oncol. 2015 Jul;24(7):1079–85.

14. Morishima T, Matsumoto Y, Koeda N, Shimada H, Maruhama T, Matsuki D, et al. Impact of Comorbidities on Survival in Gastric, Colorectal, and Lung Cancer Patients. J Epidemiol. 2019 Mar 5;29(3):110–5.

15. Jørgensen TL, Hallas J, Friis S, Herrstedt J. Comorbidity in elderly cancer patients in relation to overall and cancer-specific mortality. Br J Cancer. 2012 Mar;106(7):1353–60.

16. Moro-Sibilot D, Aubert A, Diab S, Lantuejoul S, Fourneret P, Brambilla E, et al. Comorbidities and Charlson score in resected stage I nonsmall cell lung cancer. Eur Respir J. 2005 Sep;26(3):480–6.

17. Seigneurin A, Delafosse P, Trétarre B, Woronoff AS, Velten M, Grosclaude P, et al. Are comorbidities associated with long-term survival of lung cancer? A population-based cohort study from French cancer registries. BMC Cancer [Internet]. 2018 Dec [cited 2019 Feb 13];18(1). Available from: https://bmccancer.biomedcentral.com/articles/10.1186/s12885-018-5000-7

18. Zhao L, Leung LH, Wang J, Li H, Che J, Liu L, et al. Association between Charlson comorbidity index score and outcome in patients with stage IIIB-IV non-small cell lung cancer. BMC Pulm Med. 2017 Aug 15;17(1):112.

19. Janssen-Heijnen MLG, Lemmens VEPP, van den Borne BEEM, Biesma B, Oei SB, Coebergh JWW. Negligible influence of comorbidity on prognosis of patients with small cell lung cancer: a population-based study in the Netherlands. Crit Rev Oncol Hematol. 2007 May;62(2):172–8.

20. Grønberg BH, Sundstrøm S, Kaasa S, Bremnes RM, Fløtten Ø, Amundsen T, et al. Influence of comorbidity on survival, toxicity and health-related quality of life in patients with advanced non-small-cell lung cancer receiving platinum-doublet chemotherapy. Eur J Cancer. 2010 Aug;46(12):2225–34.

21. Elixhauser A, Steiner C, Harris DR, Coffey RM. Comorbidity measures for use with administrative data. Med Care. 1998 Jan;36(1):8–27.

22. van Walraven C, Austin PC, Jennings A, Quan H, Forster AJ. A modification of the Elixhauser comorbidity measures into a point system for hospital death using administrative data. Med Care. 2009 Jun;47(6):626–33.

23. Jean RA, Chiu AS, Boffa DJ, Detterbeck FC, Blasberg JD, Kim AW. When good operations go bad: The additive effect of comorbidity and postoperative complications on readmission after pulmonary lobectomy. Surgery. 2018 Aug;164(2):294–9.

24. Tammemagi CM, Neslund-Dudas C, Simoff M, Kvale P. Impact of comorbidity on lung cancer survival. Int J Cancer. 2003 Mar 1;103(6):792–802.

25. Tammemagi CM. Comorbidity and Survival Disparities Among Black and White Patients With Breast Cancer. JAMA. 2005 Oct 12;294(14):1765.

26. Colinet B, Jacot W, Bertrand D, Lacombe S, Bozonnat MC, Daurès JP, et al. A new simplified comorbidity score as a prognostic factor in non-small-cell lung cancer patients: description and comparison with the Charlson’s index. Br J Cancer. 2005 Nov 14;93(10):1098–105.

27. Kuo YW, Jerng JS, Shih JY, Chen KY, Yu CJ, Yang PC. The Prognostic Value of the Simplified Comorbidity Score in the Treatment of Small Cell Lung Carcinoma. J Thorac Oncol. 2011 Feb;6(2):378–83.

28. Sandfeld-Paulsen B, Meldgaard P, Aggerholm-Pedersen N. Comorbidity in Lung Cancer: A Prospective Cohort Study of Self-Reported versus Register-Based Comorbidity. J Thorac Oncol. 2018 Jan;13(1):54–62.

29. Alexander M, Evans SM, Stirling RG, Wolfe R, Officer A, MacManus M, et al. The Influence of Comorbidity and the Simplified Comorbidity Score on Overall Survival in Non–Small Cell Lung Cancer—A Prospective Cohort Study. J Thorac Oncol. 2016 May;11(5):748–57.

30. Chen CY, Chen KY, Shih JY, Yu CJ. Clinical factors associated with treatment toxicity of pemetrexed plus platinum in elderly patients with non-small cell lung cancer. J Formos Med Assoc [Internet]. 2020 Jan [cited 2020 Feb 25]; Available from: https://linkinghub.elsevier.com/retrieve/pii/S0929664619311507

31. Sarfati D. Review of methods used to measure comorbidity in cancer populations: No gold standard exists. J Clin Epidemiol. 2012 Sep;65(9):924–33.
