# Supplementary figures and images for: Comparison of seven comorbidity scores on four-month survival of lung cancer patients
Source: BMC Med Res Methodol. 2023 Nov 3;23:256. doi: 10.1186/s12874-023-01994-6 (PMC10623755; doi:10.1186/s12874-023-01994-6)

**Figure S1.** Kaplan Meier curve for follow-up time in this cohort (cut off at 4 months).

**
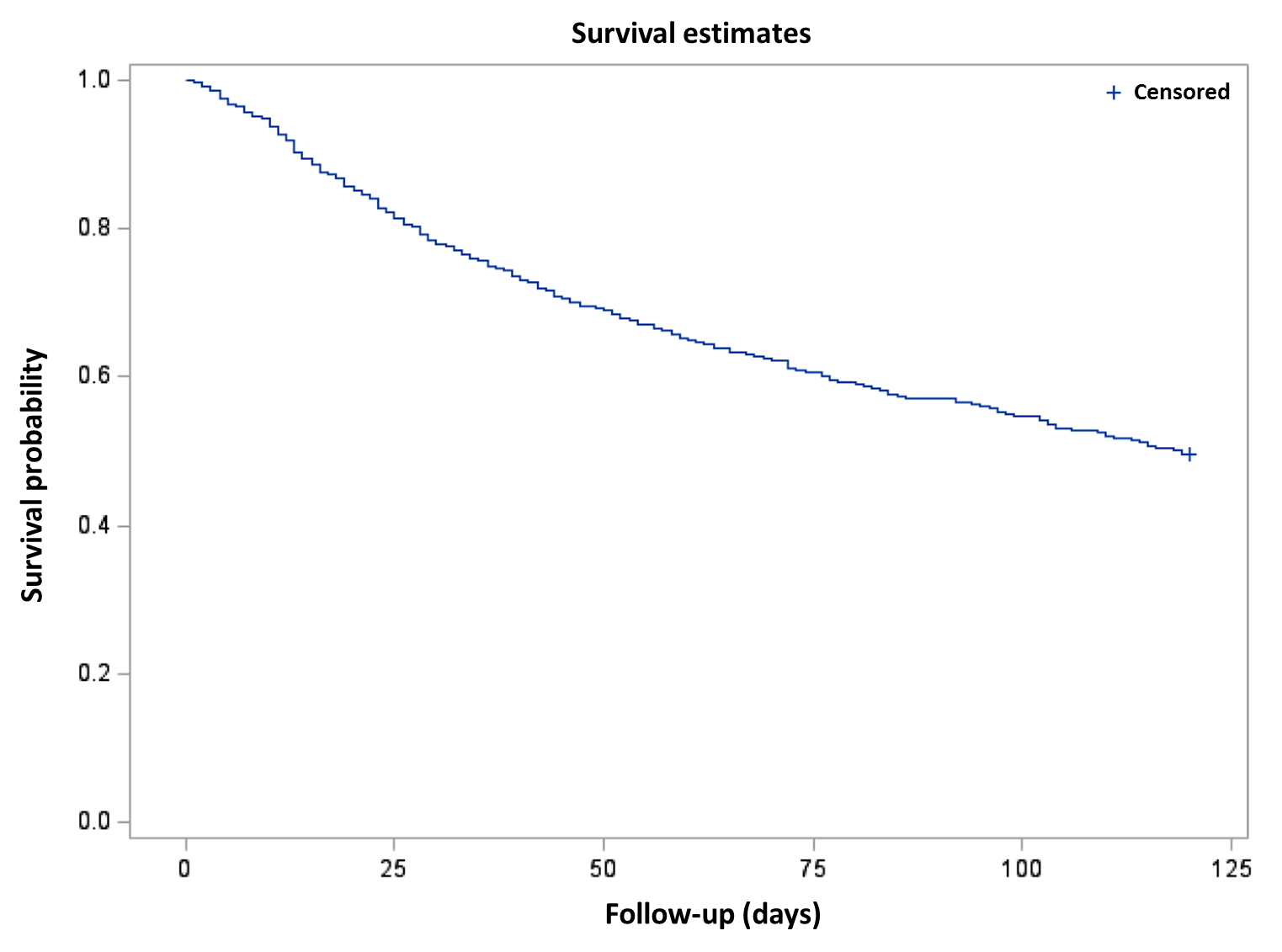
**

Supplement: Supplementary file 2 — Supplementary Material 2: Figure S1 [file 12874_2023_1994_MOESM2_ESM.docx]
