# Supplementary material for: Comparison of seven comorbidity scores on four-month survival of lung cancer patients
Source: BMC Med Res Methodol. 2023 Nov 3;23:256. doi: 10.1186/s12874-023-01994-6 (PMC10623755; doi:10.1186/s12874-023-01994-6)
Supplement: Supplementary file 3 — Supplementary Material 3: Table S2 [file 12874_2023_1994_MOESM3_ESM.docx]

**Table S2.** Description of comorbidity in the population according to CCI and CCI-lung

| Population (n=633) | CCI | CCI-lung |
| --- | --- | --- |
| Congestive heart failure | 46 (7.3) | 46 (7.3) |
| Dementia | 4 (0.6) | 4 (0.6) |
| Chronic pulmonary disease | 94 (14.9) | 94 (14.9) |
| Rheumatological disease | 4 (0.6) | 4 (0.6) |
| Mild liver disease | 12 (1.9) |  |
| Moderate or severe liver disease | 9 (1.4) |  |
| Diabetes with complications | 11 (1.7) | 11 (1.7) |
| Hemiplegia or paraplegia | 49 (7.7) | 49 (7.7) |
| Renal disease | 26 (4.1) | 26 (4.1) |
| Acute myocardial infarction |  | 4 (0.6) |
| History of myocardial infarction |  | 23 (3.6) |
| Cerebrovascular disease |  | 33 (5.2) |
| Cancer* | 65 (10.3) |  |
| AIDS | 1 (0.2) |  |

*Qualitative variables are expressed as n (%)*

**excluding lung cancer and metastatic solid tumor**s*
