# Supplementary material for: Comparison of seven comorbidity scores on four-month survival of lung cancer patients
Source: BMC Med Res Methodol. 2023 Nov 3;23:256. doi: 10.1186/s12874-023-01994-6 (PMC10623755; doi:10.1186/s12874-023-01994-6)
Supplement: Supplementary file 4 — Supplementary Material 4: Table S3 [file 12874_2023_1994_MOESM4_ESM.docx]

**Table S3.** Description of comorbidity in the population according to NCI and NCI-lung

| Population (n=633) | NCI | NCI-lung |
| --- | --- | --- |
| Acute myocardial infarction | 4 (0.6) | 4 (0.6) |
| History of myocardial infarction | 23 (3.6) | 23 (3.6) |
| Congestive Heart Failure | 46 (7.3) | 46 (7.3) |
| Peripheral Vascular disease | 48 (7.6) | 48 (7.6) |
| Cerebrovascular disease | 33 (5.2) | 33 (5.2) |
| Chronic Obstructive Pulmonary Disease | 94 (14.9) | 94 (14.9) |
| Dementia | 4 (0.6) | 4 (0.6) |
| Paralysis | 49 (7.7) | 49 (7.7) |
| Diabetes or diabetes with complications | 65 (10.3) | 70 (11.1)* |
| Renal disease | 26 (4.1) | 26 (4.1) |
| Mild liver disease or liver disease | 15 (2.4) |  |
| Peptic ulcer disease | 6 (1) | 6 (1) |
| Rheumatological disease | 4 (0.6) | 4 (0.6) |
| AIDS | 1 (0.2) |  |

*Qualitative variables are expressed as n (%)*

**in the NCI-lung score, this comorbidity is divided for diabetes (59 (9.3)) and diabetes with complications (11 (11.7)). The difference is explained by the fact that five individuals were coded with both diabetes with no complications and diabetes with complications*
