# Supplementary material for: Comparison of seven comorbidity scores on four-month survival of lung cancer patients
Source: BMC Med Res Methodol. 2023 Nov 3;23:256. doi: 10.1186/s12874-023-01994-6 (PMC10623755; doi:10.1186/s12874-023-01994-6)
Supplement: Supplementary file 5 — Supplementary Material 5: Figure S2 [file 12874_2023_1994_MOESM5_ESM.docx]

**Figure S2.** AIC, BIC, c-statistic, and Harrell’s c-statistic boxplots for the 1,000 values of each score.

CCI1: CCI, CCI2: ACCI, CCI3: CCI-lung, EX1: Elixhauser, EX2: Elixhauser-lung, NCI1: NCI, NCI2: NCI-lung
